# How Smart Technology Affects the Well-Being and Supportive Learning Performance of Logistics Employees?

**DOI:** 10.3389/fpsyg.2021.768440

**Published:** 2022-01-20

**Authors:** Fei Jiang, Li Wang, Jian-Xin Li, Jie Liu

**Affiliations:** ^1^School of Modern Circulation, Guangxi International Business Vocational College, Nanning, China; ^2^International Business Department, Shangdong College of Economics and Business, Weifang, China; ^3^Department of Computer Engineering, Dongguan Polytechnic, Dongguan, China

**Keywords:** smart technology, learning performance, well-being, self-efficacy, corporate trust

## Abstract

The rapid improvement of technologies such as artificial intelligence in recent years has resulted in the development of smart technologies (ST) that can influence learning performance in different fields. The purpose of study is to explore the link between smart technology and learning performance. Using the S-O-R model as a framework, the researchers argue that smart technology (Stimuli) will increase corporate trust, self-efficacy, and well-being (Organism), resulting in improved learning performance (Response). The current model regards corporate trust and self-efficacy as relationship factors and investigates their direct influence on employee well-being and learning performance and the mediating role played by these variables. Additionally, the function of employee well-being in moderating the relationship between corporate trust, self-efficacy, and employee learning performance is also explored. The respondents (n = 516) in the present study are made up of employees from 10 logistics companies located in China. The data analysis is conducted using the AMOS software. The results show that that smart technologies can affect learning performance through corporate trust, self-efficacy, and employee well-being. The implementation of smart technology initiatives by corporations may provide positive workplace outcomes for employees (increased well-being), corporations (more engagement in workplace learning performance), and the relationship between employees and the companies that employ them (corporate trust and self-efficacy).

## Introduction

The rapid improvement of technologies such as artificial intelligence in recent years has resulted in the development of smart technologies (ST). Such technologies not only enable significant efficiency gains in the industry but are also becoming an increasingly important competitive factor (Kuhn and Lucke, 2021). Meanwhile, smart technologies, such as artificial intelligence, act as integrative mechanisms and affect learning performance in different fields (Rane et al., 2020). Moreover, the COVID-19 pandemic has brought about significant changes in employee training, as seen in the logistics industry. More specifically, it has substantially compromised the training activities that can be provided, thus leading to a slowdown in learning performance (Prentice and Nguyen, 2020). To facilitate continuity of logistics training during such circumstances, it became necessary to implement alternative training methods using smart technologies. In practice, smart technologies such as artificial intelligence (AI) are upending corporate models and transforming how people operate around the world. For example, AI can instantaneously respond to learners' questions and provide customized replies, which boosts learning performance. At the same time, these smart technologies have a significant impact on jobs and tasks in addition to generating potential improvements in organizational efficiency (Braganza et al., 2021). In light of recent technological advancements, such as the ability to use AI technologies to improve learning performance in training provided by logistics companies, there is a clear need to fully investigate how smart technology can benefit employees in light of corporate requirements (Kaleel Ahmed et al., 2018). Meanwhile, the ubiquitous adoption of smart technologies such as AI in the workplace is likely to affect the learning performance of employees. However, very little research has been carried out in this area (Marmier et al., 2021; Arias-Pérez and Vélez-Jaramillo, 2022). This article will develop and test a model to describe how essential elements affect employees' perceptions of learning using smart technology to suit corporate needs and present the results.

The organizational literature highlights employee workplace well-being as a critical Research Topic (Erdil and Ertosun, 2011; Sadick and Kamardeen, 2020; Liu-Lastres and Wen, 2021). Some experts argue that smart technology might help us to better comprehend employee well-being (Hänsel, 2016; Papagiannidis and Marikyan, 2020; Sequeiros et al., 2021). However, at present, there is a lack of research into the influence of smart technology on employee well-being in the context of logistics training. This is somewhat puzzling as many logistics personnel have direct contact with customers, and employee well-being is associated with various outcomes that can positively influence employee attitudes toward customers and learning performance. As previously stated, a deeper knowledge of the factors that impact employees' well-being has proven to be particularly essential for this business. On this basis, there is an urgent need to resolve the issue of whether increased smart technology usage improves learning performance for employees in terms of well-being, trust and self-efficacy. Currently, few pieces of research have been carried out investigating this field, and as such, the present paper looks to address this gap.

As Erdil and Ertosun (2011) explain that when people engage with a stimulus (S), their internal state (O) is nourished, which in turn induces reactions (R) (Erdil and Ertosun, 2011; Perumal et al., 2021). In the researchers' original S-O-R model, many components of the physical environment serve as external stimuli, to which individuals can respond. They argue that the organism in the model consists of an internal structure and the process between any final reactions and external inputs, as opposed to just the final reactions themselves. Lee et al. (2011) build on this construction, posting that stimuli (such as object stimuli and social psychological stimuli) induce people's cognitive and emotional states, which in turn drive behavioral responses such as approach or avoidance (Lee et al., 2011; Lee and Min, 2021).

This study aims to look at the link between smart technology and learning performance. Using the S-O-R model as a framework, the researchers argue that smart technology (Stimuli) will cultivate corporate trust, self-efficacy, and well-being (Organism), resulting in improved learning performance (Response) (Lee et al., 2011). The current model characterizes corporate trust and self-efficacy as relationship factors and investigates their direct influence on employee well-being and learning performance, in addition to the mediating role these variables play. Additionally, the function of employee well-being in moderating the relationship between corporate trust, self-efficacy, and employee learning performance is also explored.

Taken collectively, the present research's contributions for academics and practitioners are 4 fold: Firstly, it considers previous academics' proposals to further investigate social consequences linked with smart technology activities. The study explores employee well-being and learning performance as potential social consequences of smart technology utilization and evaluates how they are related. Secondly, this study puts corporate trust and employee self-efficacy forward as key relationship variables that might serve as mediators in the context of logistics training. Thirdly, it investigates the moderating role played of employee well-being with regard to the relationship between corporate trust and employee learning performance. The theoretical contribution of this research primarily adopts the utilization of the S-O-R model as an overarching framework that improves research development.

The remainder of the paper is arranged in the following manner to understand further the effect of smart technologies on the employees' learning performance. The relevant literature is presented in section Literature Review and Hypothesis. Section Methods and Materials discusses the data as well as the empirical technique used in the study for testing the hypothesis. The findings are presented and discussed in section Empirical Analysis. Section Discussion and Conclusion provides concluding observations, while section Future Development and Limitations provide further development and limitation.

## Literature Review and Hypothesis

### Smart Technology

**Figure 1:**
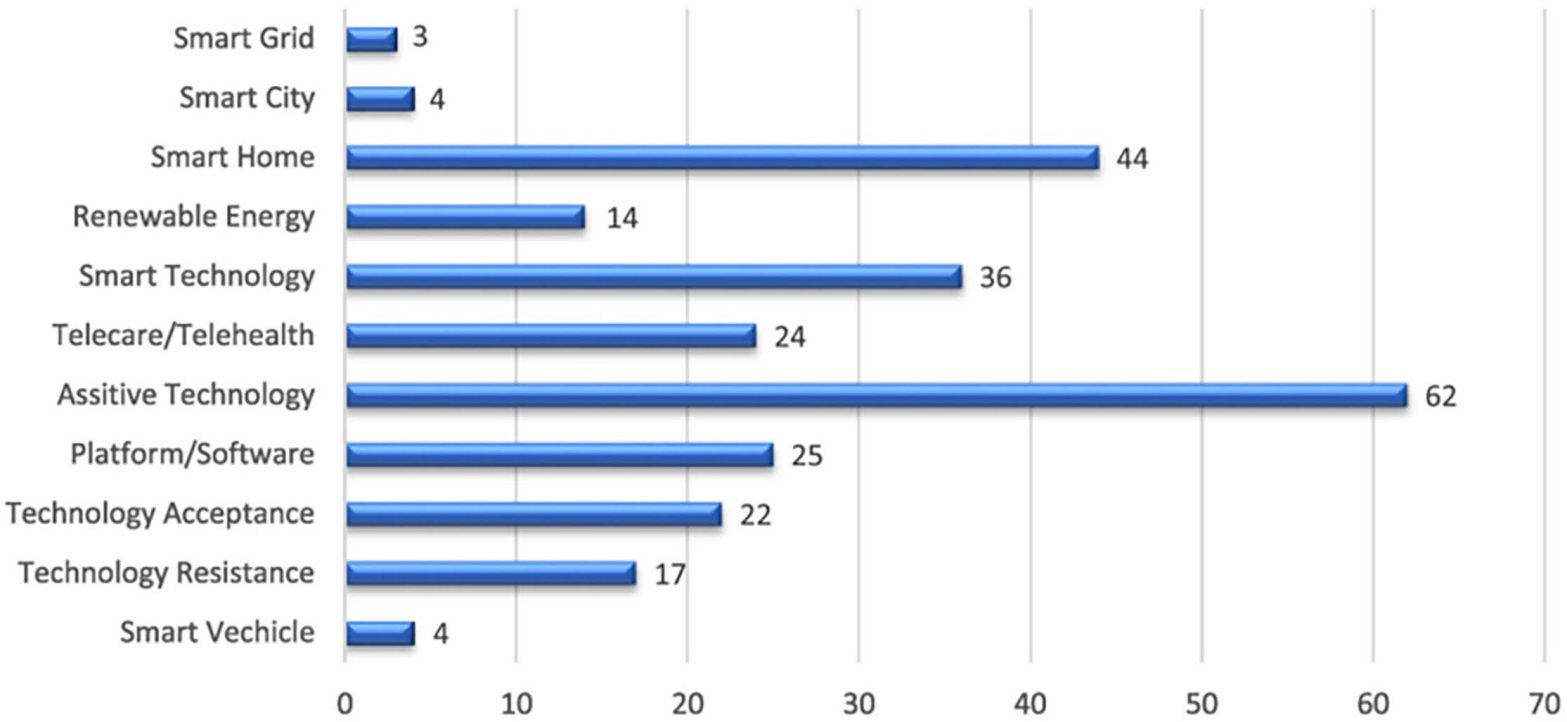
Smart technology usage (Marikyan et al., 2019).

The term “smart technology” refers to entities in which physical devices or processes are complemented with the smart properties of digital technology (Nasiri et al., 2020). In recent years, the significance of virtual reality (VR), artificial intelligence (AI), blockchain and other technologies have been widely recognized and incorporated into both operations and operational research (Choi, 2019). For example, the application of big data and AI analysis can be beneficial for companies by reducing channel costs, improving supply chain efficiency, and increasing economic value by meeting customers' changing needs. Businesses are being radically shaken up by smart technologies, which are transforming the way people all around the world are working. It has an impact on jobs and tasks whilst also having the potential to increase organizational efficiency. Furthermore, Grabowski et al. (2021) found that employees who had been trained using VR were more accurate in the completion of their corporate work and created fewer timeouts than employees instructed using non-smart approaches (Grabowski et al., 2021). Smart technology is already being used in the corporate world in various forms, such as machine learning and chatbots, amongst others. In addition, based on the systematic review of intelligent technology literature by Marikyan et al. (2019), the presence of smart technology is increasing, and it is accounting for an increasing proportion of daily life and work as Figure 1 shown (Marikyan et al., 2019).

### Corporate Trust

Throughout history, trust has helped to forge links between entities, and at the same time, it has been crucial for the maintenance of those connections (Han et al., 2021). Chen et al. (2021) view trust as consisting of two essential characteristics: (1) Belief in the other party's capabilities and (2) The willingness or inclination to depend on another party's abilities (Chen et al., 2021). According to this definition, corporate trust manifests as “individuals' expectations regarding networks of corporate relationships and behaviors.” In other words, trust is premised on the belief that future actions will be advantageous to the trusting party's situation. Notably, those employees with high levels of company trust are more likely to engage in a wide variety of work and are less likely to leave their jobs (Chauhan et al., 2021). Moreover, other academics have noted that further research into business confidence in applying smart technology is required. They insist that the entire business process will be more transparent following the integration of intelligent technologies such as smart contracts. In this way, the trust between the company and its employees will increase. In summary, according to the findings of several scholars, smart technologies influence confidence in an organization (Jayashankar et al., 2018; De Filippi et al., 2020). The following hypothesis is developed based on these previous findings:

**H1a**. Smart technology positively affects logistics employees' level of corporate trust.

### Self-Efficacy

Self-efficacy is defined as an individual's belief in their ability to complete a given activity successfully. According to Bandura's social cognition theory (1986), individuals cultivate belief in their own talents as a result of the difficulties with which they are confronted (Bandura, 1986). In this study, success is understood as the significant ability to complete a goal in a company (Gregory et al., 2021). Self-efficacy influences both direct and indirect individual performance and behavioral outcomes through its influence on tenacity, motivation, resilience, and one's capability to cope (Tramontano et al., 2021). A number of studies have reported that self-efficacy profoundly impacts academic performance in the field of educational psychology (Capron Puozzo and Audrin, 2021; Tramontano et al., 2021; Yeh et al., 2021). The ability to foresee favorable outcomes, engage in demanding tasks and maintain the dedication to learning are characteristics of self-efficacious learners, which often result in positive academic outcomes (Feldon et al., 2019).

Evolutionary psychology can provide an alternative (or additional) explanation for the potential variations in interest sparked by human learning partners and AI. Cognitive load scholars researching this field have suggested that primary psychological involvement results in lower unnecessary cognitive burdens than physiologically secondary types of interaction (Gregory et al., 2021). Meanwhile, lowering unnecessary cognitive burdens during the learning process can improve learning results. In addition to having an impact on learning results, cognitive load can also affect where an individual draws their motivation from to continue their education (e.g., self-efficacy) (Namaziandost and Çakmak, 2020).

Bandura (1986) believes that self-efficacy tends to undergo Sökmen (2020) [changes during the learning task and in different learning environments (Bandura, 1986).] investigated middle people's behavioral, emotional, cognitive, and academic engagement in science in relation to self-efficacy in Turkey, finding that self-efficacy was a positive predictor for all aspects of employee engagement (Sökmen, 2020). In this study, relative to the learning context, forms of smart learning, such as the VR technique, can be regarded as a novel learning environment (Namaziandost and Çakmak, 2020). However, to the best of our knowledge, few studies adopt the perspective of smart techniques to examine the effect of self-efficacy on employee engagement (corporate performance) in logistics training. On this basis, the following hypothesis is put forward:

**H1b**. Smart technology affects logistics employees' level of self-efficacy.

### Employee Well-Being

Based on the viewpoints put forward by Djourova et al. (2020), employee well-being includes both physical and mental elements (Djourova et al., 2020). Employee fear, tiredness, sadness, and self-esteem are all examples of mental elements, whilst headaches, lightheadedness, and gastrointestinal problems are examples of physical symptoms. As a measure of overall life satisfaction, well-being is an issue that goes far beyond simply affecting corporate members. Studies have shown that employee well-being is essential to the success of the company. For example, Singh et al. (2019) confirmed that a diminished feeling of well-being negatively affects employees' psychologically and physically, leading to higher healthcare expenditure and decreased productivity (Singh et al., 2019; Liu-Lastres and Wen, 2021). In addition, well-being also influences employees' behaviors and attitudes, such that it is necessary for companies to understand how their initiatives impact the well-being of their employees.

Bravi et al. (2018) demonstrated that digital technology could enhance the quality of employees' work-life (Bravi et al., 2018). As a result, the present article adopts the view that smart technology efforts may help to cultivate a positive work environment, leading to increased employee well-being. According to Havrda and Rakova (2020), this link to employee well-being may be especially significant when the use of smart technology aligns with workers' psychological concerns (Havrda and Rakova, 2020). The authors of this paper are unaware of any previous study examining the influence of intelligent technology on employee well-being in the logistics industry. The following hypothesis is put forward:

**H1c**. Smart technology positively affects logistics employees' well-being.

### Learning Performance

Smart technologies also provide a prime opportunity for employees to learn, which will help them become familiar with ongoing technology and software development in the market and stay up to date (Chung, 2021). It will automatically understand and provide employees with appropriate training through the analysis of documents and tests (Lee et al., 2015). According to their job descriptions, relevant skill information will be allocated to employees to better promote development. At the same time, new employees will receive training in the use of artificial intelligence and other technologies to help them quickly integrate into the new working environment. In addition, employees in non-technical roles can also use artificial intelligence training to enhance learning and improve work efficiency (Marinova et al., 2017). Based on the information, artificial intelligence in smart technology can analyze data and inform the team of the areas that employees require training in. This wise strategy will improve the efficiency of employees and provide them with faster, better quality training. In addition, smart technologies can teach specific procedures use certain teaching abilities to allow employees to self-learn and execute according to the company's needs (Tong et al., 2021). Thus, the researchers put forward the following hypothesis:

**H1d**. Smart technology positively affects logistics employees' learning performance.

Following the view of Coyle-Shapiro and Shore (2007), further study is required to determine how the employee-organization connection affects areas such as staff stress and mental well-being (Coyle-Shapiro and Shore, 2007). Meanwhile, according to Chughtai et al. (2015), employee psychological requirements are met when employees believe that their employer will fairly treat them and compensate them for their work, resulting in increased employee well-being (Chughtai et al., 2015). Trust instills confidence in a company amongst staff members and boost employees' feelings of self-efficacy. Contrastingly, low trust in a company is related to the employee's perception that they will not be treated fairly for their efforts (Jena et al., 2018). Employee well-being suffers where there is a lack of trust, which exacerbates stress and lowers job engagement levels and increases emotional weariness. Mozumder (2018) discovered that increased trust at any company level is positively connected with employee well-being (Mozumder, 2018). Based on these past results, the following hypothesis is put forward:

**H2a**. Corporate trust positively affects logistics employees' well-being.

The likelihood of employees participating in positive self-initiated discretionary acts outside the bounds of their employment contract increases when they believe their organizations are trustworthy in their judgement (Yoon et al., 2016). According to research, corporate trust is positively associated with constructive social activities, particularly organizational citizenship behaviors (Hendriks et al., 2020). Corporate trust acts to motivate employees. Employees are motivated by corporate trust (Chiang and Hsieh, 2012). According to social exchange theory, employees will invest more effort into an organization if they trust it. They will be more likely to participate in good learning performance in particular. Yoon et al. (2016) discovered a link between company trust and employee behavior in the context of logistics. (Yoon et al., 2016). Researchers have not identified any previous research investigating the direct relationship between logistics employee learning performance and corporate trust. Hence, the hypothesis can be designed as follows:

**H2b**. Corporate trust positively affect logistics employee learning performance.

Employee well-being is often investigated as part of organizational stress research, emphasizing the effects of workplace factors such as technology on employee learning (Djourova et al., 2020). Theoretical and empirical evidence point to the importance of self-efficacy for employee well-being. When it comes to employee well-being, emotional weariness and work satisfaction are regarded to be important markers, according to organizational literature (Sabri et al., 2020). Emotional exhaustion is a term that refers to the state of mental and physical tiredness that occurs as a result of the progressive depletion of an individual's energetic resources at work. It is regarded to be a fundamental element of burnout (Huang et al., 2019). Generally speaking, work satisfaction is related to employees' emotional assessments of their jobs, and it is seen as a predictor of employees' subjective well-being (Liu et al., 2010). Employee withdrawal from work is influenced by various factors, including emotional fatigue and job satisfaction (Singh et al., 2019). Based on prior research, the researchers hypothesized that self-efficacy might be a significant predictor of employee well-being. Employees who are more confident in their ability to perform in the future reported less emotional tiredness and higher levels of job satisfaction than employees who are less confident in their future effectiveness. As a result, the following theory is put forth:

**H3a**. Self-efficacy positively affects employee well-being.

There has been little research done to explain the direct relationship between self-efficacy and learning performance conceptually. Existing research supports the notion that self-efficacy impacts the degree to which individuals strive for learning success. Some scholars have identified that boosting self-efficacy is beneficial to improving learning performance, especially in using smart devices (Parschau et al., 2013; Kostagiolas et al., 2019; Sun and Hsu, 2019). Consequently, the researcher suggests that various interpretations of the process and its impact on work performance may arise due to this inadequacy (Chen, 2017). For instance, Tims et al. (2014) suggested that individuals with a strong sense of self-efficacy in a particular field are more likely to stay on the job for a more extended period and to be more self-regulated to deal with the challenges of the job, which, in turn, contributes to better overall job performance (Tims et al., 2014).

Much empirical research primarily supports the positive link between self-efficacy and work performance, despite a lack of solid theoretical explanations for the relationship and the subsequent controversy over these explanations in the literature (Chen, 2017). Tims et al. (2014) observed that workers who felt more self-efficacious were more likely than their disengaged peers to attain improved job performance (Tims et al., 2014). It should be noted that the empirical data shown above is mostly linked to a more generic sense of self-efficacy, as opposed to task/job-specific self-efficacy. Furthermore, existing research suggests that task/job-specific self-efficacy has a significantly more significant association with work performance when compared to more broad self-efficacy (e.g., Quiñ, 1997; Stajkovic and Luthans, 1998; Chen, 2017). Therefore, the researcher will predict that task-specific (e.g., logistics) self-efficacy will positively connect with work performance (learning performance in our case).

**H3b**. Self-efficacy positively affects employee learning performance.

The well-being and motivation of two components of life can be easily connected. Imagine how many employees seek Internet inspiration. Workers who cannot find any drive might be comfortable. What's the connection between the two? Think about things that are motivating. For many, the profession learns something new or sophisticated. Continuous personnel training is a huge motivator and helps wellness (Khoreva and Wechtler, 2018). Better education has to do in a few ways with well-being. It maintains the intellect sharp first. Learning new things helps battle memory issues and illnesses such as Alzheimer's, even late in life (Wu et al., 2020). New knowledge can connect directly to the well-being of employees. For example, employees may be provided with job stress or anxiety management (Kaminitz, 2020). The time to attend such a course all day long may be stimulating and beneficial to the well-being of employees. Other forms of motivation may occur in team-building exercises. A range of activities from sports to trivia night adds a totally new level of well-being to the social component, but there are still opportunities that promote well-being. Communication and social contact may often help improve workers' morale and skills (Yen and Lin, 2020). In addition, staff in the logistic company may discover that working together is considerably easier by familiarizing themselves with staff other than their typical departmental obligations. Further seamless work with a good bureau culture. This not only increases the degree of motivation across the board but also dramatically reduces stress levels. Discourage negative remarks and make work happier to observe actual changes in the performance of the employees in the logistic industry (Lin and Sironi, 2020). This not only improves the stress level of the office but may also increase human well-being in general. Authors claim in this study that employee well-being is how the employee learns. The welfare of workers affects the productivity and success of their companies. The improvement of well-being enhances the performance of employees in logistics and lowers non-certified claims for sick leave, damages and compensation. Thus, the authors hypothesize:

**H4**. Employees well-being positively affects learning performance.

**Figure 2:**
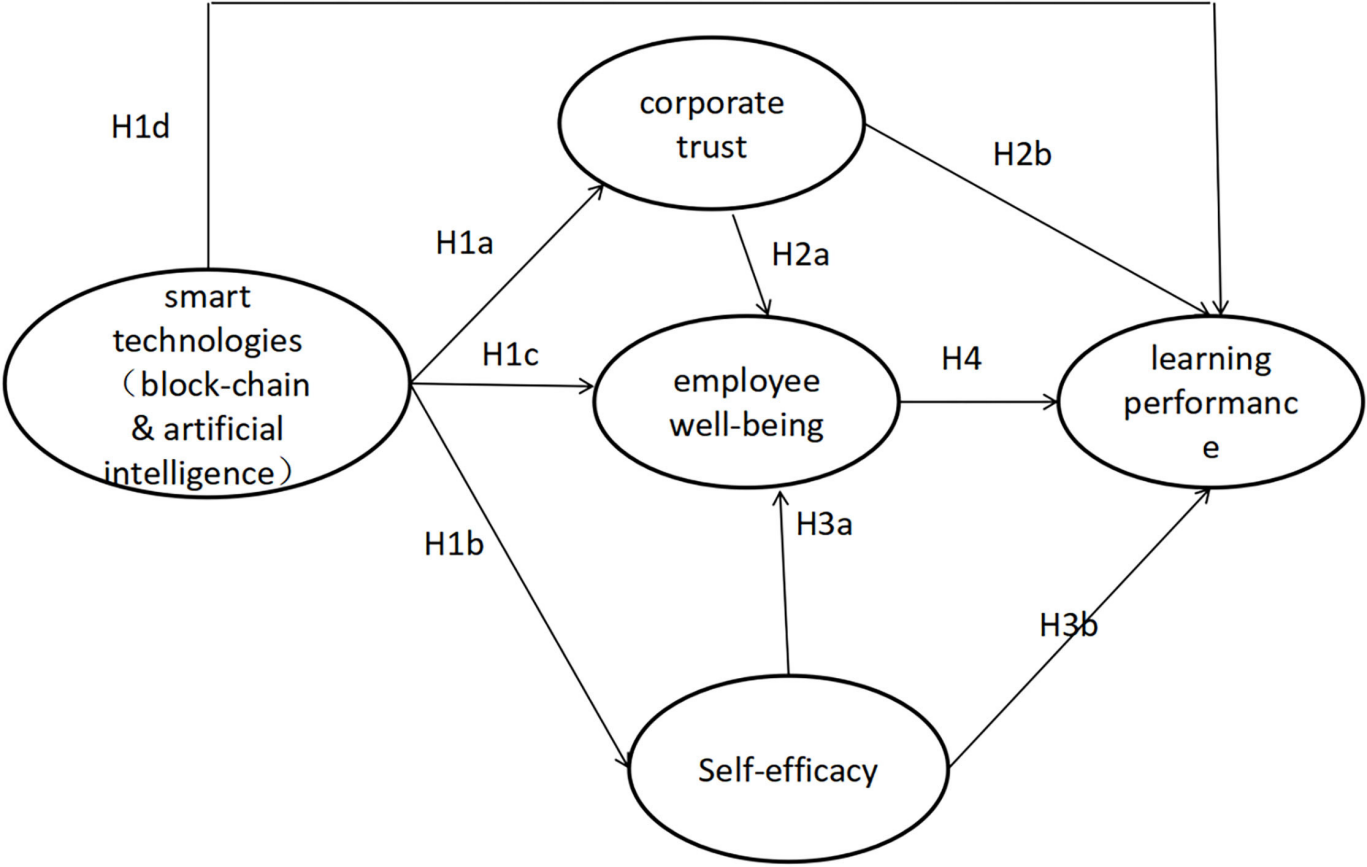
Conceptual framework.

Following the hypotheses, the conceptual model (Figure 2) can represent the network of relationships among smart technologies and learning performance, corporate trust, employee well-being, and self-efficacy.

## Methods and Materials

### Measures

All items in the research questionnaire were used from existing studies. A seven-point Likert scale was used to evaluate each question, with the lowest value indicating “strongly disagree” (1), and the highest value indicating “strongly agree” (7). Six academic native English speakers assessed the back-translation for conceptual equivalence and how the newest version replicated the original text (Debets et al., 2020). Subsequently, the procedure can be repeated with 10 Chinese logistics management academics who had not been engaged in the initial creation of the questionnaire. After forward translation, they offered feedback on the questionnaire's design and the phrasing of scale elements. Ultimately, an acceptable questionnaire version was reached by two rounds of back-translations based on the comments received.

### Pretest of the Measures

The researcher invited 60 logistics workers who had worked in logistics companies with relevant logistics training to participate in a pretest for ensuring that all items were easily comprehended by the target demographic of interest. The researcher can optimize the questionnaire based on their suggestions by format, length, wording, and sequence. These workers did not take part in the survey's final administration. Pretest responders agreed to answer all survey questions and report any difficulties or concerns during a debriefing. Each item had a standard factor loading of more than 0.500 (*p* < 0.001), and all scales had a Cronbach's Alpha better than 0.80.

### Collecting Data

The study has used a purposive sample approach for conducting empirical research. Despite the restrictions associated with generalization problems, purposive sampling is a practical and appropriate method when the researcher should concern respondents with specific characteristics and experience to better assist with related surveys, as in the case of a questionnaire survey on a specific topic (Etikan, 2016). The influence of smart technology on employees' learning performance could not be approved without referencing particular logistics companies and asking for employees in the assessment survey for assessing the employees (Elsbach and Bhattacharya, 2001). Therefore, a particular corporate was referenced on the evaluation survey for assessing the logistics employees. The corporation was selected after conducting an in-depth evaluation of a prominent China-based logistics corporates with a large number of employees and is also a well-known brand in the logistics sector.

Over 10 weeks, the Analysis was performed at ten famous logistics companies such as Shun Feng Group Co., Ltd., Deepon Logistics Co., Ltd. and tec., which are excellent Chinese logistics companies with one-stop integrated logistics solutions. The questionnaires were delivered to staff by the researchers in collaboration with the managers at each company. The potential respondents were not asked for any identifying information, and they were given a blank envelop in which to return their finished questionnaire. The sealed envelope was then placed in a safe box, which was gathered several days after the data collection procedure began at participating companies by one of the researchers. According to Frohlich (2002), researchers have contacted respondents by phone or email before and after the questionnaire was issued to improve the recovery rate (Frohlich, 2002). The poll was kept anonymous to prevent supervisors from learning how an individual staff member answered. Five hundred and thirty-fiveof the 600 issued surveys were returned, 516 of which contained finished questionnaires. All respondents worked in existing positions for more than 2 years, accounting for 98.6 and 87%. Therefore, the authenticity and validity of the questionnaires' data can be guaranteed.

### Data Analysis

Structural equation modeling (SEM) was used to assess measurement and structural models in this research. The SEM technique shines out when analyzing a complicated research model with small samples and nonnormalized data (Gefen and Straub, 2005). In particular, it can support the research model testing, including second-order reflective constructs as well as formative constructs. In this study, technology readiness is characterized as a second-order formative construct. According to the above explanation, the study uses this technique to validate measurement models and research hypotheses. A structural model should be developed once the measurement model has been thoroughly analyzed (Hoc et al., 2014). Measurement models are used to verify the discriminant and convergent validity of items and constructs, whilst the structural models confirm that the hypothesized links in the research model are correct (Hoc et al., 2014; Hair et al., 2019; Chang and Chen, 2021).

## Empirical Analysis

**Figure 3:**
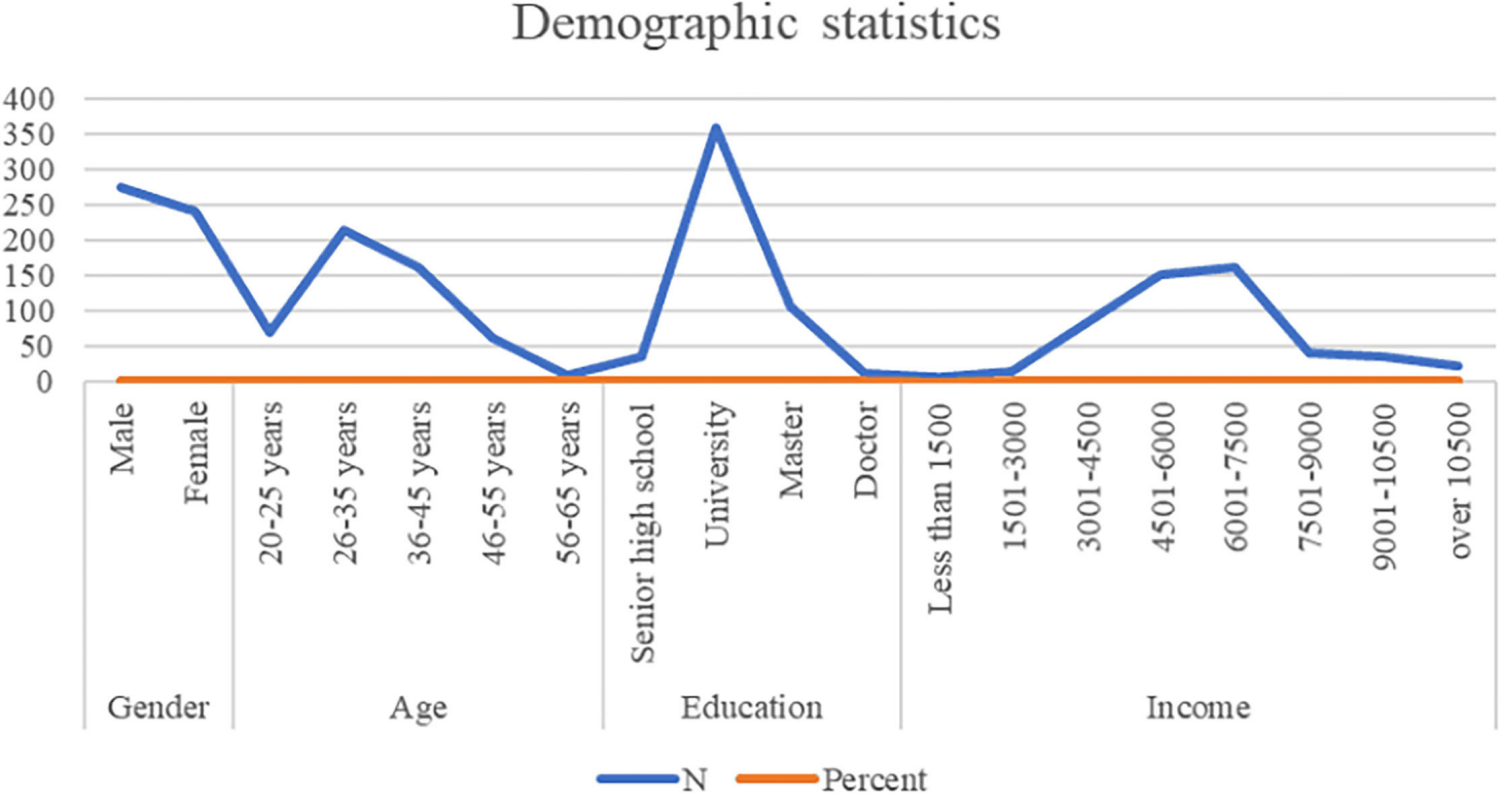
Demographic.

The empirical survey was conducted in Shun Feng Group, and researchers cooperated with Group to collect data from employees. According to the demographic data shown in Figure 3, 375 of participants in the survey were predominantly male (72.67%), 216 were between 26 and 35 years old (41.86%), 361 had university degrees (69.96%), and 162 had monthly incomes between RMB 6,001 and 7,500 (31.40%).

### Multivariate Normality Test

Researchers checked the data for multivariate normality before evaluating the measurement model to ensure that the SEM assumptions could be met. Current results show the absolute values of univariate skewness and kurtosis, which were <2.0 and <3.0, respectively. Hence, results were not varied much from the normal distribution.

### Common Method Variance Test

Self-assessment questionnaires are prone to common method deviation problems, and common method deviation tests need to be carried out. There are many methods, each with its own advantages and disadvantages. The advantage of Harman's single factor test is that it is simple and easy to be used in this research. An exploratory factor analysis was performed using the SPSS software to incorporate all of the measuring items. The solution discovered a total of five variables. The total amount of variance that could be explained was 77.13%. According to the eigenvalue of the component with the greatest eigenvalue, it explained 37.16 % of the total variance, which was less than predicted.

### Measurement Model

According to calculation, all constructs' Cronbach's alpha has exceeded 0.700. The composite reliabilities (CR) varied between 0.889 and 0.937. All factor loadings were more than 0.500 and statistically significant (*p* = 0.001). The square of the root of AVE values (0.694–0.835) exceeds correlation values (0.240–0.637), reflecting the satisfactory discriminant validity, as Table 1 shows.

**Table 1:** The correlation.

	**1**	**2**	**3**	**4**	**5**
1.Smart technology	**0.835**				
2. Corporate trust	0.45	**0.694**			
3. Self-efficacy	0.381	0.691	**0.797**		
4. Employee well-being	0.365	0.67	0.593	**0.784**	
5. Learning performance	0.339	0.649	0.612	0.61	**0.801**

*The AVE values were bolded*.

### Structural Model With Testing

The structural path model's fitting indices demonstrate an acceptable overall fit to the data. Table 2 shows that smart technology significantly affects corporate trust, self-efficacy, employee well-being, and learning performance. The Hypothesis 1 subtypes are all supported. Company trust in the logistics company shows a direct and statistically significant link with employee well-being as well as learning performance, giving support for both H2a and H2b. Employee well-being and learning performance are both positively influenced by their self-efficacy with the business. H3a and H3b are upheld in their entirety. Findings from the study suggest that employee well-being has a favorable impact on learning performance, which supports Hypothesis 4.

**Table 2:** Measurement outcomes.

**Hypothesized relationships**	**Path label**	**Standard path loadings**	***T*** **-value**	**Standard error**	**Hypothesis test outcome**
H1a smart technology → corporate trust	λ21	0.178^c^	3.639	0.43	Yes
H1b smart technology → self-efficacy	λ31	0.257^c^	5.148	0.59	Yes
H1c smart technology → employee well-being	λ41	0.089^a^	1.957	0.45	Yes
H1d smart technology → learning performance	λ51	0.124^b^	3.009	0.42	Yes
H2a corporate trust → employee well-being	β42	0.198^c^	4.299	0.51	Yes
H2b corporate trust → learning performance	β52	0.179^c^	4.061	0.49	Yes
H3a self-efficacy → employee well-being	β43	0.418^c^	8.219	0.40	Yes
H3b self-efficacy → learning performance	β53	0.459^c^	8.878	0.41	Yes
H4 employee well-being → learning performance	β54	0.198^c^	3.457	0.50	Yes

a *p < 0.05*.

b *p < 0.01*.

c *p < 0.001*.

### Effects

Table 3 offers information on the relationships between the constructs and their impacts (direct, indirect, and total). Self-efficacy has the greatest direct influence on employee well-being out of the three antecedents studied. In addition, it was shown that self-efficacy had the largest direct impact on learning performance. This study discovered that smart technology had a significant indirect influence on employee well-being and workplace learning performance. Self-efficacy had the largest overall influence on learning performance (directly and indirectly through employee well-being).

**Table 3:** Hypothesis outcomes.

**Hypothesized relationships**	**Direct effects**	**Indirect effects**	**Total effects**
Smart technology → corporate trust	0.319		0.319
Smart technology → self-efficacy	0.292		0.292
Smart technology → employee well-being	0.104	0.108779	0.327
Smart technology → learning performance	0.11	0.095381	0.342
Organizational trust → employee well-being	0.391		0.391
Organizational trust → learning performance	0.199	0.094622	0.294
Self-efficacy → employee well-being	0.341		0.341
Self-efficacy → learning performance	0.299	0.082522	0.381
Employee well-being → learning performance	0.242		0.242

## Discussion and Conclusion

Previous research on the impacts of smart technologies on employees has chiefly focused on the psychological effects of the technology. To provide only a few examples: work satisfaction (Oh et al., 2017). The authors' findings contribute to the knowledge of the crucial role that the employee-company connection plays in explaining the influence of smart technology on two critical employee social outcomes. Crucially, the findings of the current study have consequences for managerial practice.

The current study's findings have implications for managerial practice. Firstly, one approach to encouraging workers to participate in behaviors that promote employee learning efforts is to ensure that the company's smart technology usage policies are clearly stated to all employees and managers. Staff members should be aware of the company's efforts to foster learning and hold employees accountable for their use of smart technology, stressing the importance of taking an active role in those efforts. It is necessary to utilize a range of smart technologies to guide the company's action, as some authors have pointed out in their articles (Foroudi et al., 2018; Lebioda et al., 2019; Langley et al., 2021). To be informed about the diversity, value, and success of smart technology usage in the business, they must have access to readily available information. As our data indicate, providing employees with access to smart technology in the workplace will significantly increase their sense of trust and self-efficacy.

The emergence of smart technologies has prompted an important transformation in the logistics industry. Researchers have made the case that the activities undertaken in establishing trust and self-efficacy with the employing business through learning performance centered on smart technology use are critical. To their credit, many companies are now dedicating significant resources to improving learning performance and establishing a variety of social welfare projects. Researchers believe that how a company and its employees employ smart technologies is essential for both the organization and its personnel. To better leverage the development of employee relationships and the subsequent positive results linked with smart technology, organizations should support initiatives that foster a sense of belonging to the organization amongst employees.

Finally, research has found that that employee well-being might directly impact the performance of employees in terms of their learning. In addition to the correlations investigated in the present study, logistics managers may deploy various techniques to promote employee well-being in their organizations. For example, investments in psychology should be made by establishing a workplace environment that encourages the use of smart technologies to increase interpersonal and technical abilities and staff productivity. An organization may create settings that support workers to enhance their skill sets while also supporting their sense of security, acceptability, and well-being through the use of smart technologies to accomplish these objectives.

## Future Development and Limitations

The present research's findings contribute to the existing literature on smart technologies and learning. It also raises additional questions for future research to address. Firstly, smart technology is a multi-dimensional construct that is composed of various technologies and can be used in the logistics industry. Smart technology was assessed and examined as a single construct, despite each of these aspects being represented in the current study. There is space to investigate the function played by each dimension in the model and explore the possible implications of each smart technology dimension on the subsequent variables, especially with regard to the mediator effect. In recent years, researchers have increasingly been adopting more robust ways to statistically test the mediation effect. However, they still require a robust approach to generating mediation hypotheses and interpreting research results (Rasoolimanesh et al., 2021). Therefore, future research can consider studying the intermediary variables of this model. This study assessed the relationship between self-efficacy and corporate trust to illustrate the learning performance across different logistics companies and their respective workers. On the basis of the findings detailed here, we urge further investigation into the influence of smart technologies on the preservation of learning outcomes in the logistics context. It should be noted that the current study's findings, which polled logistics employees in central China, may not be applicable in other contexts as the sample size was small. Future studies may wish to test the supplied model in a different country to further evaluate the generalisability of the findings presented here.

## Data Availability Statement

The original contributions presented in the study are included in the article/Supplementary Material, further inquiries can be directed to the corresponding author/s.

## Ethics Statement

The studies involving human participants were reviewed and approved by Ningbo University Ethics Committee. The patients/participants provided their written informed consent to participate in this study. Written informed consent was obtained from the individual(s) for the publication of any potentially identifiable images or data included in this article.

## Author Contributions

All authors listed have made a substantial, direct, and intellectual contribution to the work and approved it for publication.

## Funding

This work was supported by 2020 Guangxi Vocational Education supports this paper and Teaching Reform Key Research Project Under the background of health and wealth planning, the four-in-one mixed teaching research and practice of class match and post certificate for financial majors (GXGZJG2020A012).

## Conflict of Interest

The authors declare that the research was conducted in the absence of any commercial or financial relationships that could be construed as a potential conflict of interest.

## Publisher's Note

All claims expressed in this article are solely those of the authors and do not necessarily represent those of their affiliated organizations, or those of the publisher, the editors and the reviewers. Any product that may be evaluated in this article, or claim that may be made by its manufacturer, is not guaranteed or endorsed by the publisher.

## References

[B1] Arias-Pérez J. Vélez-Jaramillo J. (2022). Ignoring the three-way interaction of digital orientation, Not-invented-here syndrome and employee's artificial intelligence awareness in digital innovation performance: a recipe for failure. Technol. Forecast. Soc. Change 174:121305. 10.1016/j.techfore.2021.121305

[B2] Bandura A. (1986). Social Foundations of Thought and Action: A Social Cognitive Theory. New Jersey, NJ: Prentice-Hall.

[B3] Braganza A. Chen W. Canhoto A. Sap S. (2021). Productive employment and decent work: the impact of AI adoption on psychological contracts, job engagement and employee trust. J. Business Res. 131, 485–494. 10.1016/j.jbusres.2020.08.018 32836565 PMC7434459

[B4] Bravi L. Murmura F. Santos G. (2018). Manufacturing labs: Where new digital technologies help improve life quality. Int. J. Qual. Res. 12, 957–974. 10.18421/IJQR12.04-11

[B5] Capron Puozzo I. Audrin C. (2021). Improving self-efficacy and creative self-efficacy to foster creativity and learning in schools. Thinking Skills Creat. 42:100966. 10.1016/j.tsc.2021.100966

[B6] Chang Y. W. Chen J. (2021). What motivates customers to shop in smart shops? The impacts of smart technology and technology readiness. J. Retail. Consumer Serv. 58:102325. 10.1016/j.jretconser.2020.102325

[B7] Chauhan Y. Jaiswall M. Goyal V. (2021). Does societal trust affect corporate capital structure? Emerg. Markets Rev. 8, 1–15. 10.1016/j.ememar.2021.100845

[B8] Chen I. S. (2017). Computer self-efficacy, learning performance, and the mediating role of learning engagement. Comput. Hum. Behav. 72, 362–370. 10.1016/j.chb.2017.02.059

[B9] Chen Z. Chen F. Zhou M. (2021). Does social trust affect corporate environmental performance in China? Energy Econ. 102:105537. 10.1016/j.eneco.2021.105537

[B10] Chiang C. F. Hsieh T. S. (2012). The impacts of perceived organizational support and psychological empowerment on job performance: the mediating effects of organizational citizenship behavior. Int. J.of Hosp. Manag. 31, 180–190. 10.1016/j.ijhm.2011.04.011

[B11] Choi T. M. (2019). Blockchain-technology-supported platforms for diamond authentication and certification in luxury supply chains. Transport. Res. Part E Logist. Transport. Rev. 128, 17–29. 10.1016/j.tre.2019.05.011

[B12] Chughtai A. Byrne M. Flood B. (2015). Linking ethical leadership to employee well-being: the role of trust in supervisor. J. Business Ethics 128, 653–663. 10.1007/s10551-014-2126-7

[B13] Chung S. (2021). Applications of smart technologies in logistics and transport : a review. Transport. Res. Part E 153:102455. 10.1016/j.tre.2021.102455

[B14] Coyle-Shapiro J. A. M. Shore L. M. (2007). The employee-organization relationship: where do we go from here? Hum. Resour. Manag. Rev. 17, 166–179. 10.1016/j.hrmr.2007.03.008

[B15] De Filippi P. Mannan M. Reijers W. (2020). Blockchain as a confidence machine: the problem of trust and challenges of governance. Technol. Soc. 62:101284. 10.1016/j.techsoc.2020.101284

[B16] Debets M. P. M. Scheepers R. A. Boerebach B. C. M. Arah O. A. Lombarts K. M. J. M. H. (2020). Variability of residents' ratings of faculty' s teaching performance measured by five- and seven-point response scales. BMC Med Educ. 20:325. 10.1186/s12909-020-02244-9 32962692 PMC7510269

[B17] Djourova N. P. Rodríguez Molina I. Tordera Santamatilde N. Abate G. (2020). Self-efficacy and resilience: mediating mechanisms in the relationship between the transformational leadership dimensions and well-being. J. Leadership Organiz. Stud. 27, 256–270. 10.1177/1548051819849002

[B18] Elsbach K. D. Bhattacharya C. B. (2001). Defining who you are by what you're not: organizational disidentification and the national rifle association. Organiz. Sci. 12, 393–413. 10.1287/orsc.12.4.393.10638

[B19] Erdil O. Ertosun Ö. G. (2011). The relationship between social climate and loneliness in the workplace and effects on employee well-being. Proc. Soc. Behav. Sci. 24, 505–525. 10.1016/j.sbspro.2011.09.091

[B20] Etikan I. (2016). Comparison of convenience sampling and purposive sampling. Am. J. Theoret. Appl. Stat. 5, 1–4. 10.11648/j.ajtas.20160501.1124899564

[B21] Feldon D. F. Callan G. Juth S. Jeong S. Learning S. (2019). Cognitive load as motivational cost. Educ. Psychol. Rev. 31, 319–337. 10.1007/s10648-019-09464-6

[B22] Foroudi P. Gupta S. Sivarajah U. Broderick A. (2018). Investigating the effects of smart technology on customer dynamics and customer experience. Comput. Hum. Behav. 80, 271–282. 10.1016/j.chb.2017.11.014

[B23] Frohlich M. T. (2002). Techniques for improving response rates in OM survey research. J. Operat. Manag. 20, 53–62. 10.1016/S0272-6963(02)00003-7

[B24] Gefen D. Straub D. (2005). A practical guide to factorial validity using PLS-graph: tutorial and annotated example. Commun. Assoc. Inform. Syst. 16, 91–109. 10.17705/1CAIS.01605

[B25] Grabowski A. Jankowski J. Wodzyński M. (2021). Teleoperated mobile robot with two arms: the influence of a human-machine interface, VR training and operator age. Int. J. Hum. Comput. Stud, 156:102707. 10.1016/j.ijhcs.2021.102707

[B26] Gregory L. R. Ramjan L. M. Villarosa A. R. Rojo J. Cl M. N. Raymond D.. (2021). Does self-ef fi cacy for medication administration predict clinical skill performance in fi rst-year nursing students? An inception-cohort study. Teach. Learn. Nurs. 34, 1–17. 10.1016/j.teln.2021.10.002

[B27] Hair J. F. Risher J. J. Sarstedt M. Ringle C. M. (2019). When to use and how to report the results of PLS-SEM. Eur. Business Rev. 31, 2–24. 10.1108/EBR-11-2018-0203

[B28] Han W. Huang Y. Hughes M. Zhang M. (2021). The trade-off between trust and distrust in supply chain collaboration. Indus. Market. Manag. 98, 93–104. 10.1016/j.indmarman.2021.08.005

[B29] Hänsel K. (2016). Wearable and ambient sensing for well-being and emotional awareness in the smart workplace, in UbiComp 2016 Adjunct - Proceedings of the 2016 ACM International Joint Conference on Pervasive and Ubiquitous Computing (London: ACM), 411–416. 10.1145/2968219.2971360

[B30] Havrda M. Rakova B. (2020). Enhanced well-being assessment as basis for the practical implementation of ethical and rights-based normative principles for AI, in IEEE Transactions on Systems, Man, and Cybernetics: Systems (Toronto, ON: IEEE), 2754–2761. 10.1109/SMC42975.2020.9283137

[B31] Hendriks M. Rijsenbilt A. Pleeging E. Commandeur H. (2020). Virtuous leadership: a source of employee well-being and trust. Manag. Res. Rev. 43, 951–970. 10.1108/MRR-07-2019-0326

[B32] Hoc L. Fong N. Law R. (2014). A Primer on partial least squares structural equation modeling (PLS-SEM). Eur. J. Tourism Res. 6, 211–213. 10.1080/1743727X.20

[B33] Huang S. Yin H. Lv L. (2019). Job characteristics and teacher well-being: the mediation of teacher self-monitoring and teacher self-efficacy. Educ. Psychol. 39, 313–331. 10.1080/01443410.2018.1543855

[B34] Jayashankar P. Nilakanta S. Johnston W. J. Gill P. Burres R. (2018). IoT adoption in agriculture: the role of trust, perceived value and risk. J. Business Indus. Market. 33, 804–821. 10.1108/JBIM-01-2018-0023

[B35] Jena L. K. Pradhan S. Panigrahy N. P. (2018). Pursuit of organisational trust: role of employee engagement, psychological well-being and transformational leadership. Asia Pac. Manag. Rev. 23, 227–234. 10.1016/j.apmrv.2017.11.001

[B36] Kaleel Ahmed A. Senthilkumar C. B. Nallusamy S. (2018). Study on environmental impact through analysis of big data for sustainable and green supply chain management. Int. J. Mech. Prod. Eng. Res. Dev. 8, 1245–1254. 10.24247/ijmperdfeb2018145

[B37] Kaminitz S. C. (2020). Looking good or feeling well? Understanding the combinations of well - being indicators using insights from the philosophy of well - being. Soc. Indicat. Res. 12, 1–16. 10.1007/s11205-020-02289-9

[B38] Khoreva V Wechtler H. (2018). HR practices and employee performance: the mediating role of well-being. Employee Relat. 40, 227–243. 10.1108/ER-08-2017-019124938460

[B39] Kostagiolas P. Lavranos C. Korfiatis N. (2019). Learning analytics: survey data for measuring the impact of study satisfaction on students' academic self-efficacy and performance. Data Brief 25:104051. 10.1016/j.dib.2019.104051 31211203 PMC6562179

[B40] Kuhn C. Lucke D. (2021). ScienceDirect ScienceDirect Supporting the Digital Transformation : a low-threshold approach for supporting low-threshold CIRP transformation : Nantes, France Approach for Manufacturing Related Higher Education and Employee Training Manufacturing Related. Proc. CIRP 104, 647–652. 10.1016/j.procir.2021.11.109

[B41] Langley D. J. van Doorn J. Ng I. C. L. Stieglitz S. Lazovik A. Boonstra A. (2021). The internet of everything: smart things and their impact on business models. J. Business Res. 122, 853–863. 10.1016/j.jbusres.2019.12.035

[B42] Lebioda L. Hahn I. S. MattosMartins A. A. (2019). The influence of mobile technology usage behavior on perceived work performance improvement. Int. J. Dev. Res. 9, 25733–25738.

[B43] Lee J. Choi M. Lee H. (2015). Factors affecting smart learning adoption in workplaces: comparing large enterprises and SMEs. Inform. Technol. Manag. 16, 291–302. 10.1007/s10799-014-0201-5

[B44] Lee S. K. Min S. R. (2021). Effects of information quality of online travel agencies on trust and continuous usage intention: an application of the SOR model. J. Asian Finan. Econ. Business 8, 971–982. 10.13106/jafeb.2021.vol8.no4.0971

[B45] Lee Y. K. Kim Y. S. Son M. H. Lee D. J. (2011). Do emotions play a mediating role in the relationship between owner leadership styles and manager customer orientation, and performance in service environment? Int. J. Hosp. Manag. 30, 942–952. 10.1016/j.ijhm.2011.02.002

[B46] Lin J. Sironi E. (2020). Logistic maximum likelihood estimation for optimal well-being determinants. IEEE Trans. Emerg. Topics Comput. 9, 1316–1327. 10.1109/TETC.2020.300929527295638

[B47] Liu J. Siu O. L. Shi K. (2010). Transformational leadership and employee well-being: the mediating role of trust in the leader and self-efficacy. Appl. Psychol. 59, 454–479. 10.1111/j.1464-0597.2009.00407.x

[B48] Liu-Lastres B. Wen H. (2021). How do ethnic minority foodservice workers perceive employee well-being? An exploratory study. J. Hosp. Tourism Manag. 46, 376–383. 10.1016/j.jhtm.2021.01.013

[B49] Marikyan D. Papagiannidis S. Alamanos E. (2019). A systematic review of the smart home literature: a user perspective. Technol. Forecast. Soc. Change 138, 139–154. 10.1016/j.techfore.2018.08.015

[B50] Marinova D. de Ruyter K. Huang M. H. Meuter M. L. Challagalla G. (2017). Getting smart: learning from technology-empowered frontline interactions. J. Serv. Res. 20, 29–42. 10.1177/1094670516679273

[B51] Marmier F. Deniaud I. Rasovska I. Michalak J.-L. (2021). Towards a proactive vision of the training for the 4.0 Industry: from the required skills diagnostic to the training of employees. IFAC PapersOnLine 54, 1144–1149. 10.1016/j.ifacol.2021.08.135

[B52] Mozumder N. A. (2018). A multilevel trust-based model of ethical public leadership. J. Business Ethics 153, 167–184. 10.1007/s10551-016-3341-1

[B53] Namaziandost E. Çakmak F. (2020). An account of EFL learners' self-efficacy and gender in the Flipped Classroom Model. Educ. Inform. Technol. 25, 4041–4055. 10.1007/s10639-020-10167-7

[B54] Nasiri M. Ukko J. Saunila M. Rantala T. (2020). Managing the digital supply chain: the role of smart technologies. Technovation 96–97:02121. 10.1016/j.technovation.2020.102121

[B55] Oh S. Lee J. M. Kim Y. (2017). A study on the job satisfaction in the smart work *Environment* 8, 393–401. 10.15207/JKCS.2017.8.11.393

[B56] Papagiannidis S. Marikyan D. (2020). Smart offices: a productivity and well-being perspective. Int. J. Inform. Manag. 51:102027. 10.1016/j.ijinfomgt.2019.10.012

[B57] Parschau L. Fleig L. Koring M. Lange D. Knoll N. Schwarzer R.. (2013). Positive experience, self-efficacy, and action control predict physical activity changes: a moderated mediation analysis. Br. J. Health Psychol. 18, 395–406. 10.1111/j.2044-8287.2012.02099.x 23013288

[B58] Perumal S. Ali J. Shaarih H. (2021). Exploring nexus among sensory marketing and repurchase intention: application of S-O-R Model. Manag. Sci. Lett. 11, 1527–1536. 10.5267/j.msl.2020.12.020

[B59] Prentice C. Nguyen M. (2020). Engaging and retaining customers with AI and employee service. J. Retail. Consumer Serv. 56:102186. 10.1016/j.jretconser.2020.102186

[B60] Quiñ M. A. (1997). Running head: Meta-Analysis: Self-Efficacy and Performance. Houston: Annual conference of the Society.

[B61] Rane S. B. Thakker S. V. Kant R. (2020). Stakeholders' involvement in green supply chain: a perspective of blockchain IoT-integrated architecture. Manag. Environ. Qual. Int. J. 32, 1–25. 10.1108/MEQ-11-2019-0248

[B62] Rasoolimanesh S. M. Wang M. Roldán J. L. Kunasekaran P. (2021). Are we in right path for mediation analysis? Reviewing the literature and proposing robust guidelines. J. Hosp. Tourism Manag. 48, 395–405. 10.1016/j.jhtm.2021.07.013

[B63] Sabri M. F. Wijekoon R. Rahim H. A. (2020). The influence of money attitude, financial practices, self-efficacy and emotion coping on employees' financial well-being. Manag. Sci. Lett. 10, 889–900. 10.5267/j.msl.2019.10.007

[B64] Sadick A. M. Kamardeen I. (2020). Enhancing employees' performance and well-being with nature exposure embedded office workplace design. J. Build. Eng. 32:101789. 10.1016/j.jobe.2020.101789

[B65] Sequeiros H. Oliveira T. Thomas M. A. (2021). The impact of IoT smart home services on psychological well-being. Inform. Syst. Front. 3, 1–18. 10.1007/s10796-021-10118-8

[B66] Singh S. K. Pradhan R. K. Panigrahy N. P. Jena L. K. (2019). Self-efficacy and workplace well-being: moderating role of sustainability practices. Benchmarking 26, 1692–1708. 10.1108/BIJ-07-2018-0219

[B67] Sökmen Y. (2020). The role of self-efficacy in the relationship between the learning environment and student. Educ. Stud. 47, 19–37. 10.1080/03055698.2019.166598629298942

[B68] Stajkovic A. D. Luthans F. (1998). Self-efficacy and work-related performance: a meta-analysis. Psychol. Bull. 124, 240–261. 10.1037/0033-2909.124.2.240

[B69] Sun J. C. Y. Hsu K. Y. C. (2019). A smart eye-tracking feedback scaffolding approach to improving students' learning self-efficacy and performance in a C programming course. Comput. Hum. Behav. 95, 66–72. 10.1016/j.chb.2019.01.036

[B70] Tims M. Bakker A. B. Derks D. (2014). Daily job crafting and the self-efficacy – performance relationship. J. Manager. Psychol. 29, 490–507. 10.1108/JMP-05-2012-0148

[B71] Tong S. Jia N. Luo X. Fang Z. (2021). The Janus face of artificial intelligence feedback: deployment versus disclosure effects on employee performance. Strat. Manag. 42, 1600–1631. 10.1002/smj.332225855820

[B72] Tramontano C. Grant C. Clarke C. (2021). Development and validation of the e-Work Self-Efficacy Scale to assess digital competencies in remote working. Comput. Hum. Behav. Rep. 4:100129. 10.1016/j.chbr.0.2021.100129 34568639 PMC8452465

[B73] Wu H. Li S. Zheng J. Guo J. (2020). Medical students' motivation and academic performance: the mediating roles of self-efficacy and learning engagement. Med. Educ. Online 25:1742964. 10.1080/10872981.2020.1742964 32180537 PMC7144307

[B74] Yeh C. H. Lin H. H. Wang Y. M. Wang Y. S. Lo C. W. (2021). Investigating the relationships between entrepreneurial education and self-efficacy and performance in the context of internet entrepreneurship. Int. J. Manag. Educ. 19:100565. 10.1016/j.ijme.2021.100565

[B75] Yen W. Lin H. (2020). Investigating the effect of flow experience on learning performance and entrepreneurial self- efficacy in a business simulation systems context. Interact. Learn. Environ. 3, 1–16. 10.1080/10494820.2020.1734624

[B76] Yoon D. Jang J. Lee J. H. (2016). Environmental management strategy and organizational citizenship behaviors in the hotel industry: the mediating role of organizational trust and commitment. Int. J. Contemp. Hosp. Manag. 28, 1577–1597. 10.1108/IJCHM-10-2014-0498

