# Supplementary material for: How Smart Technology Affects the Well-Being and Supportive Learning Performance of Logistics Employees?
Source: Front Psychol. 2022 Jan 20;12:768440. doi: 10.3389/fpsyg.2021.768440 (PMC8810824; doi:10.3389/fpsyg.2021.768440)
Supplement: Supplementary file 1 [file Table_1.DOCX]

Appendix I

Questionnaire Survey for Smart Technology Application to Learning Performance

| The purpose of this questionnaire is to investigate the smart technology application in company especially in learning performance, self-efficacy, well-being, corporate trust. There is no right or wrong answer in this survey. Your answer will be kept strictly confidential. Please answer according to the actual situation. |
| --- |
| Personal information:  Gender: ____ A. male. B. female  Age: ____ A. 20-25 years B.26-35 years  C.36-45 years D.46-55 years E.56-65 years  Education:____ A.Senior high school B. University C. Master D. Doctor  Income (RMB): ____ A. Less than 1500 B.1501-3000 C.3001-4500  D.4501-6000 E.6001-7500 F.7501-9000  G.9001-10500 H.over 10500 |
| 1. The smart technologies such as the virtual reality (VR), artificial intelligence (AI), block chain and etc.have been effectively used in workplace.___   1. Strongly agree B. Agree C. More or less agree D. Undecided   E. More or less disagree F.Strongly disagree  2. The smart technologies such as the virtual reality (VR), artificial intelligence (AI), block chain can create comfortable work environment.___  A.Strongly agree B. Agree C. More or less agree D. Undecided  E. More or less disagree F.Strongly disagree  3. The smart technologies such as the virtual reality (VR), artificial intelligence (AI), block chain can affect the trust from company.___  A.Strongly agree B. Agree C. More or less agree D. Undecided  E. More or less disagree F.Strongly disagree  4.The smart technologies such as the virtual reality (VR), artificial intelligence (AI), block chain can affect the employees well-being.___  A.Strongly agree B. Agree C. More or less agree D. Undecided  E. More or less disagree F.Strongly disagree  5.The smart technologies such as the virtual reality (VR), artificial intelligence (AI), block chain can affect the self-efficacy of employees.___  A.Strongly agree B. Agree C. More or less agree D. Undecided  E. More or less disagree F.Strongly disagree  6. We trust that company can resolve social problems in an ethical manner with effective smart technology.___  A.Strongly agree B. Agree C. More or less agree D. Undecided  E. More or less disagree F.Strongly disagree  7. We trust that advanced levels of corporate trust result in a variety of positive employee citizenship behaviors and reduced turnover intentions___  A.Strongly agree B. Agree C. More or less agree D. Undecided  E. More or less disagree F.Strongly disagree  8. We trust that positive impact of smart technologies application on organizational trust.___  A.Strongly agree B. Agree C. More or less agree D. Undecided  E. More or less disagree F.Strongly disagree  9. We trust that the high level of trust on company can affect the employees well-being.___  A.Strongly agree B. Agree C. More or less agree D. Undecided  E. More or less disagree F.Strongly disagree  10. We trust that the high level of trust on company can affect our learning performance.___  A.Strongly agree B. Agree C. More or less agree D. Undecided  E. More or less disagree F.Strongly disagree  11. The poor sense of well-being will impact employees physically and psychologically, resulting in increased health insurance costs and lower worker productivity.___  A.Strongly agree B. Agree C. More or less agree D. Undecided  E. More or less disagree F.Strongly disagree  12. Employee well-being also impacts employees’ attitudes and behaviors by the application of smart technologies.___  A.Strongly agree B. Agree C. More or less agree D. Undecided  E. More or less disagree F.Strongly disagree  13. Smart technologies initiatives can aid in creating a positive work environment, which in turn leads to greater employee well-being.___   1. Strongly agree B. Agree C. More or less agree D. Undecided   E. More or less disagree F.Strongly disagree  14. Employee well-being can affect our learning performance by the application of smart technologies.___  A.Strongly agree B. Agree C. More or less agree D. Undecided  E. More or less disagree F.Strongly disagree   1. Employee well-being can reduce the employees turnover rate.___   A.Strongly agree B. Agree C. More or less agree D. Undecided  E. More or less disagree F.Strongly disagree  16. We believe they are capable of achieving task demands under various situations. ___  A.Strongly agree B. Agree C. More or less agree D. Undecided  E. More or less disagree F.Strongly disagree  17. self-efficacy can affect learning performance on the application of smart technologies___  A.Strongly agree B. Agree C. More or less agree D. Undecided  E. More or less disagree F.Strongly disagree  18.  The high self-efficacy can act as a protective barrier against stress, anxiety, or depression of employees.___  A.Strongly agree B. Agree C. More or less agree D. Undecided  E. More or less disagree F.Strongly disagree  19. self-efficacy can affect employees well-being___  A.Strongly agree B. Agree C. More or less agree D. Undecided  E. More or less disagree F.Strongly disagree  20. Learning performance can be affected by the application of smart technologies ___  A.Strongly agree B. Agree C. More or less agree D. Undecided  E. More or less disagree F.Strongly disagree  Note: Please fill in the answer in "()". |

# Appendix II

**Demographic**

| Measure | Item | N |
| --- | --- | --- |
| Gender | Male | 275 |
|  | Female | 241 |
| Age | 20-25 years | 69 |
|  | 26-35 years | 216 |
|  | 36-45 years | 161 |
|  | 46-55 years | 61 |
|  | 56-65 years | 9 |
| Education | Senior high school | 36 |
|  | University | 361 |
|  | Master | 106 |
|  | Doctor | 13 |
| Income | Less than 1500 | 7 |
|  | 1501-3000 | 15 |
|  | 3001-4500 | 83 |
|  | 4501-6000 | 151 |
|  | 6001-7500 | 162 |
|  | 7501-9000 | 41 |
|  | 9001-10500 | 36 |
|  | over 10500 | 21 |
